# The Change of Central Vein Oxygen Saturation Level during Spontaneous Breathing Trial as a Predictor of Successful Extubation in Intensive Care Unit

**DOI:** 10.2174/0118743064363511250304050459

**Published:** 2025-03-20

**Authors:** Made Wirga Wirgunatha, Bianca Jeanne, Tjokorda Gde Agung Senapathi, Putu Agus Surya Panji, I. Wayan Suranadi

**Affiliations:** 1 Department of Anesthesiology, Pain Management, and Intensive Care, Faculty of Medicine, Universitas Udayana Ngoerah General Hospital, Denpasar, Indonesia

**Keywords:** Central venous oxygen saturation, Mechanical ventilation, Spontaneous breathing trial, Successful extubation, Weaning, Intensive Care Unit

## Abstract

**Background:**

Weaning from mechanical ventilation is an essential step in the care of critically ill patients. Central venous oxygen saturation (ScvO_2_) could reflect tissue oxygenation.

**Objective:**

The evaluation of the difference in ScvO_2_ values at the beginning and end of the Spontaneous Breathing Trial (SBT) can be used as a predictor of successful extubation in critically ill patients.

**Methods:**

This cross-sectional study was conducted in the ICU of Prof. Dr. I.G.N.G. Ngoerah Hospital from July to August 2024. This study involved 42 adult patients aged 18-65 who were using mechanical ventilation during their admission to the ICU. All patients had central venous access, were clinically ready for mechanical ventilation weaning, and could attempt SBT for 30-120 minutes with a rapid shallow breathing index (RSBI) of less than 105.

**Results:**

All patients underwent 30-120 minutes of SBT. ScvO_2_ levels were measured at the beginning of SBT (first minute) and at 30 minutes after SBT started, and the change in ScvO_2_ level was recorded (ΔScvO_2_). Patients with RSBI < 105 measured during SBT were extubated. Extubation failure was defined as the need for re-intubation, or patients died within 48 hours after extubation. Of 42 patients, 37 patients (89.1%) were successfully extubated. There was a significant difference in ΔScvO_2_ between successfully extubated patients and those who failed  (-2.89±1.63 *vs.* -8.2±4.27; p=0.049). The ROC curve analysis showed that a decrease in ScvO_2_ ≤4.5% was the most optimal cut-off for a predictor of successful extubation with a sensitivity of 81.1%, specificity of 60%, positive predictive value of 93.8% and negative predictive value of 70%.

**Conclusion:**

The difference in ScvO_2_ between the beginning and after 30 minutes of SBT was a valuable early predictor for successful extubation from mechanical ventilation.

## 1 INTRODUCTION

Mechanical ventilation is essential in managing critically ill patients in the intensive care unit (ICU). However, prolonged use increases the risk of complications such as ventilator- associated pneumonia, tracheal injury, stress ulcers, and sepsis, leading to higher morbidity and mortality [1]. Timely weaning is crucial, and predicting successful weaning poses a significant clinical challenge.

To determine readiness for weaning, a spontaneous breathing trial (SBT) is performed using a T-tube with minimal pressure support, assessing the patient’s ability to breathe independently after the acute phase of illness resolves. Success in SBT is evaluated through hemodynamic stability, mental status, and oxygenation efficiency. However, a successful SBT does not guarantee successful extubation, with failure rates as high as 30% in the first 2-3 days. More accurate predictors of extubation success are needed [1, 2].

The Rapid Shallow Breathing Index (RSBI) is the most commonly used parameter for predicting extubation success due to its simplicity and high sensitivity. An RSBI below 105 breaths/min/L is a common threshold indicating likely extubation success. However, a recent meta-analysis revealed moderate sensitivity (83%) but low specificity (58%), highlighting the need for additional predictive tools [2, 3].

Global tissue oxygenation status during SBT is another potential predictor. Increased breathing effort raises oxygen demand, reducing tissue oxygenation. Mixed venous oxygen saturation (SvO_2_) and central venous oxygen saturation (SvO_2_) are key indicators. While SvO_2_ is more representative, it is invasive. SvO_2_, requiring only a central venous catheter, is less invasive and more practical. Studies suggest that greater decreases in SvO_2_ during SBT may indicate extubation failure, while smaller decreases suggest success. Despite this, SvO_2_ is not widely used due to limited data on its optimal cut-off values and prognostic accuracy [1, 2].

This study aims to evaluate whether changes in SvO_2_ during SBT can predict successful extubation in ICU patients.

## 2 METHODS

### 2.1 Study Design and Setting

This analytical observational cross-sectional study involved medical and surgical patients who were admitted to the ICU of Prof. Dr I.G.N.G. Ngoerah Hospital from July to August 2024. This study was reviewed and approved by Ethics Committee at the Faculty of Medicine Universitas Udayana (Approval Number: 1878/UN14.2.2.VII.14 /LT/2024). Informed consent was obtained from each participant or their legal guardian regarding their involvement in this study.

### 2.2 Study Participant

Adult patients aged 18-65 who were using mechanical ventilation during their admission to the ICU were involved in this study. All patients with central venous catheter access in the superior cava vein, were clinically ready for weaning, and could perform SBT for 30-120 minutes with an RSBI <150. Patients were considered clinically ready for weaning if they met these criteria:

a) Resolution of the acute phase of the disease that becomes the main indication for intubation and mechanical ventilation.

b) Adequate cough reflex (peak cough expiratory flow >60 L/min) without excessive bronchial secretions (suction was not needed every 2-3 hours).

c) Hemodynamically stable (no signs and symptoms of myocardium ischemia, systolic blood pressure within 90-160 mmHg without or with the use of minimal vasoactive support, and a heart rate ≤140 beats/min)

d) Adequate oxygenation (partial pressure of oxygen (PaO_2_) ≥60 mmHg at inspired oxygen fraction (FiO_2_) ≤40%; or ratio of PaO2/FiO_2_ >150 mmHg; positive end-expiratory pressure (PEEP) ≤8 cmH_2_O; and PaCO_2_ was within normal range or approaching patients’ baseline level).

e) Adequate pulmonary function (maximum inspiratory pressure (MIP) ≤ -20 cmH_2_O and respiratory rate ≤35 breaths/min).

f) No significant electrolyte imbalance (pH 7.35-7.45) with a hemoglobin level ≥10 g/dL.

g) Controlled pain with a behavioral pain scale (BPS) or numerical rating scale (NRS) less than 3.

h) Adequate consciousness (Glasgow Coma Scale (GCS) ≥13).

Patients who were using tracheostomy, had lung cancer or chronic obstructive pulmonary disorder (COPD), or died before weaning process, were excluded. Eligible patients were selected using consecutive sampling.

### 2.3 Study Protocol and Measurement

Each patient who was deemed ready for weaning underwent SBT for at least 30 minutes. SBT was performed using a T-piece tube with minimal pressure support (PS) ventilation mode (PS 6-8 cm H_2_O and PEEP 4 cmH_2_O), and FiO_2_ was adjusted to be the same as when the patient was using mechanical ventilation. RSBI was calculated during SBT by dividing the respiratory rate by the tidal volumes (L). The patient was extubated if RSBI <105 breaths/min/L. If the RSBI >105 breaths/min/L, the patient was not extubated, and SBT was repeated the following day. A blood sample was obtained from the central venous catheter to determine the ScvO_2_ level through blood gas analysis. ScvO_2_ level measurement was performed at the beginning of SBT (first minute) and 30 minutes after SBT started. The change in ScvO_2_ level was recorded as ΔScvO_2_. Other hemodynamic parameters, including mean arterial pressure (MAP), heart rate (HR), respiratory rate (RR), and peripheral oxygen saturation (SaO_2_), were also recorded before and after SBT. The signs of extubation failure were observed for the next 48 hours. Extubation failure was defined as the need for re-intubation, or patients died within 48 hours after extubation.

### 2.4 Statistical Analysis

A descriptive statistical analysis was carried out. Continuous data were expressed as means and standard deviations (SD), while categorical data were expressed as relative frequency (%). Baseline characteristics, ScvO_2_ pre-SBT, ScvO_2_ 30-min after SBT, and ΔScvO_2_ were compared between successfully extubated patients and those who failed. A comparative test for continuous data was performed using an independent T-test or Mann-Whitney-U test, as appropriate. Meanwhile, comparative tests for categorical data were performed using the Chi-square or Fisher exact test, as appropriate. A Receiver Operating Curve (ROC) analysis was conducted to obtain the optimal cut-off of ΔScvO_2_ for predicting successful extubation. Statistical significance was defined as p < 0.05. Diagnostic analysis was conducted to determine the diagnostic accuracy of ΔScvO_2_ in predicting successful extubation based on the optimal cut-off obtained from ROC analysis. All statistical analyses in this study were performed using Statistical Package for Social Science (SPSS) version 25.0 software.

## 3 RESULTS

Forty-two eligible patients were involved in this study, with a mean age of 42.2±15.34 years. The majority of patients (37/42; 89.1%) were successfully extubated. Detailed demographic and clinical baseline characteristics are shown in Table **1**. There was no significant difference in baseline characteristics between successfully extubated patients and those who failed, except for higher survival status in successfully extubated patients (100% *vs.* 60%, p=0.012).

There was a significant difference in RR, SaO_2_, and MAP between successfully extubated patients and those who failed at the first minute and after 30 minutes of SBT (Table **2**). The baseline PaO_2_ and ScvO_2_ were also not significantly different between successfully extubated patients and those who failed. However, when the change of PO_2_ level (ΔPaO_2_) and change of ScvO_2_ level (ΔScvO_2_) after 30 minutes of SBT were compared between groups, patients who were success- fully extubated significan- tly had a lower decrease of PaO_2_ (ΔPaO_2_ -2.54±1.88 vs -8.6±9,42; p=0.016) and ScvO_2_ (ΔScvO_2_ -2.89±1.63 vs -8.20±4.27; p=0.049) (Table **3**). Thus, a decrease in ScvO_2_ could be used to discriminate successfully extubated patients from patients more likely to fail.

**Fig. (1):**
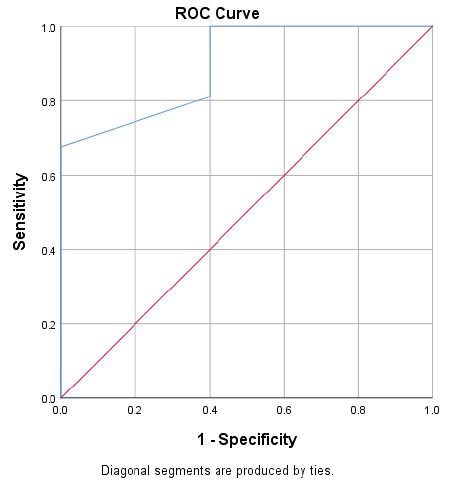
Receiver operating curve (ROC) analysis of ΔScvO_2_ for predicting successful extubation.

Further analysis using the ROC curve (Fig. **1**) showed that the most optimal ΔScvO_2_ cut-off to predict successful extubation in critically ill patients was 4.5%. Thus, a decrease of ScvO_2_ less than 4.5% is associated with a higher probability of successful extubation (Table **4**). This cut-off had an area under curve (AUC) of 0.897, sensitivity of 81.1%, specificity of 60%, positive predictive value (PPV) of 93.8%, and negative predictive value (NPV) of 70% (Table **5**).

## 4 DISCUSSION

A decreased ScvO_2_ level by less than 4.5% during SBT could predict successful extubation with high sensitivity (81.1%) and moderate specificity (60.0%). Both groups also had comparable comorbidities, mechanical ventilation duration, and even similar blood gas analysis and other laboratory parameters at baseline. This indicated that the change in ScvO_2_ level during SBT could discriminate successfully extubated patients from patients more likely to fail even if they had similar clinical characteristics. Thus, our study suggests that a decrease in ScvO_2_ level of less than 4.5% during SBT could be utilized in clinical practice as a guide for the mechanical ventilation weaning process in critically ill patients.

Previous studies also proved that ΔScvO_2_ during SBT could predict successful extubation in difficult-to-wean patients [4]. Ashmawi *et al.* also reported that a decrease of ScvO_2_ of more than 3.8% is significantly associated with extubation failure. Even though their cut-off was slightly different, this study showed that a decrease in ScvO_2_ level had a high sensitivity (89.74%), specificity (90.91%), and PPV (97.22%) but with moderate NPV (71.43%) [5]. Mallat *et al.* also found that ΔScvO_2_ was an independent predictor of extubation success in patients with good SBT tolerance. A decrease of ScvO_2_ ≥5.4% had an excellent NPV and a moderate PPV in predicting extubation failure [6]. Teixeira *et al.* also found that a greater decrease in ScvO_2_ level during a T-tube trial could predict extubation failure in 86% of cases. Meanwhile, patients who were successfully extubated had minimal change in ScvO_2_ level [7]. They found that the extubation failure and re-intubation requirement rate significantly increased when ScvO_2_ decreased to more than 4.5% (sensitivity 88%, specificity 95%, PPV 0.93, and NPV 0.90) [8]. Shalaby *et al.* also stated that the risk of re-intubation was significantly higher when the decrease in ScvO_2_ level >5% (sensitivity 87%, specificity 90%, PPV 0.78, and NPV 0.93) [7].

Oxygen consumption (VO_2_) depends on the delivery of oxygen (DO_2_) and oxygen extraction ratio (O_2_ER). Therefore, increased DO_2_ will increase VO_2_ levels. During the transition from mechanical ventilation to spontaneous breathing, the respiratory muscle will work harder, increasing VO_2_ [9, 10]. Thus, CO and DO_2_ will also increase as compensation to offset the increased demand for respiratory muscle VO_2_. Increased VO_2_ demand during this transition process will cause decreased O_2_ supply to cells, resulting in decreased global tissue oxygenation muscle [11-13]. In addition to the effects of increased VO_2_, other factors, such as laryngeal oedema and low muscle endurance, are some of the potential causes for extubation failure, which are not directly related to oxygen consumption and use, so these results may be, to some extent, difficult to explain [4]. These confounding factors (mental status alteration and inadequate cough) were excluded from our study.

The respiratory rate, MAP and SaO_2_, in the successful and unsuccessful group during the first minute and after 30 minutes show a significant difference statistically, but clinically it’s difficult to precisely predict the failure of extubation using only a small difference in respiratory rate (16.30±3.09 *vs.* 20.20±4.27), SaO_2_ (99.7±0.62 *vs.* 98.20±2.17) and MAP (84.14±11.86 *vs.* Line 187-19196.40±12.22). An Intensivist should use not one but several parameters, from patient’s vital signs, clinical conditions, and laboratory parameters that show tissue perfusion to predict the failure of extubation.

ScvO_2_ is used as a surrogate of SvO_2_, which is more invasive, high-risk, and expensive because it requires pulmonary catheter insertion. Now, ScvO_2_ has become part of standard care for critically ill patients. The previous study showed a significant correlation between ScvO_2_ and SvO_2_ [14, 15]. Therefore, ScvO_2_ measurement is also a reliable indicator for global tissue oxygenation status, but it is easier, safer, and affordable in clinical practice.

The outcomes of successfully extubated patients and patients who failed significantly differed. A higher survival rate was found in groups of successfully extubated patients (100% *vs.* 60%, p=0.012). This emphasizes that proper timing of extubation in patients ready for weaning is critical, and failure of extubation significantly affects the morbidity and mortality of critically ill patients. Therefore, we highly suggest using the change in ScvO_2_ level as a guide to predict successful extubation in critically ill patients because successful SBT does not always mean successful extubation.

This study also had limitations. It was a non-randomized study with a limited number of participants, which may limit the generalization of its results. However, it involved both surgical and medical critically ill patients. Another limitation is the absence of hemodynamic measurements to assess CO and also no SvO_2_ measurement as a gold standart test, due to the limited use of right heart catheterization. Nevertheless, the results of this study still add important points regarding the role of ScvO_2_ measurements during SBT to guide clinicians in selecting patients who are likely to be successful or unsuccessfully extubated.

## CONCLUSION

The change of ScvO_2_ during SBT was a good predictor of successful intubation in critically ill patients in the ICU. A decrease of ScvO_2_ less than 4.5% after 30 minutes of SBT is associated with a higher probability of successful extubation, with a sensitivity of 81.1%, specificity of 60%, positive PPV of 93.8%, and NPV of 70%. An Intensivist should use not one, but several parameters, from patient’s vital signs, clinical condition, laboratory parameters that show tissue perfusion to predict the failure of extubation. We suggest using this new parameter as an additional judgment in selecting patients in the ICU who are likely to be successfully extubated.

## Figures and Tables

**Table 1:** Baseline characteristics.

**Characteristics**	**All Patients (n=42)**	**Groups**		**P-value**
		**Successful Extubation (n=37)**	**Fail Extubation (n=5)**	
Age (years), mean ± SD	42.2±15.34	40.7±15.52	53.2±8.64	0.15^b^
**Sex, n (%)**				
Male	24 (57.1)	23 (62.2)	1 (20.0)	0.146^c^
Female	18 (42.9)	14 (37.8)	4 (80.0)	
BMI (kg/m^2^), mean ± SD	23.66±5.5	23.20±3.74	27.08±12.93	0.541^a^
**Diagnosis groups**				
Surgical	39 (92.9)	35 (94.6)	4 (80.0)	0.323^c^
Non-surgical	3 (7.1)	2 (5.4)	1 (20.0)	
**Comorbidities, n (%)**				
Neurologic disease	16 (38,1)	13 (35.1)	3 (60.0)	0.352^c^
Heart disease	23 (54.8)	21 (56.8)	2 (40.0)	0.644^c^
Respiratory disease	3 (7.1)	2 (5.4)	1 (20.0)	0.323^c^
Renal disease	6 (14.3)	4 (10.8)	2 (40.0)	0.141^c^
Diabetes mellitus	4 (9.5)	4 (10.8)	0 (0)	1.000^c^
Mechanical ventilation duration (days), mean ± SD	1.98±1.55	1.89±1.47	2.6±2.07	0.346^b^
Length of stays (days), mean ± SD	4.05±1.99	3.92±1.96	5±2.34	0.252^b^
**Outcome, n (%)**				
Survive or improved	40 (95.2)	37 (100)	3 (60)	0.012^c^
Died	2 (4.8)	0 (0)	2 (40)	
Laboratories parameters, mean ± SD				
Leukocyte (x10^3^/µL)	14.73±4.95	15.27±4.86	10.72±3.97	0.055^b^
Hemoglobin (g/dL)	11.1±2.01	11.01±1.97	11.76±2.46	0.44^a^
Creatinine	0.97±0.51	1.01±0.53	0.74±0.29	0.145^b^
Na	141.5±5.35	141.4±5.61	142.4±2.97	0.701^a^
K	3.85±0.74	3.92±0.74	3.33±0.56	0.098^a^
Arterial blood gas analysis, mean ± SD				
pH	7.377±0.072	7.375±0.072	7.394±0.079	0.594^a^
PO_2_	133.5±36.86	136,6±36.8	110.6±31.5	0.107^b^
PCO_2_	44.88±21.17	44.95±22.52	44.4±5.55	0.252^b^
BE	-0.12±4.66	-0.44±4.65	2.24±4.54	0.232^a^
HCO_3_	24.93±3.9	24.64±3.9	27.12±3.52	0.185^a^
SO_2_	98.48±1.45	98.65±1.36	97.2±1.64	0.072^b^

^a^Independent T-test; ^b^Mann-Whitney-U test. BE, base excess; BMI, body mass index; HCO_3_, bicarbonate; K, potassium; Na, sodium; PCO_2_, partial pressure of carbon dioxide; PO_2_, partial pressure of oxygen; SD, standard deviation; SO_2_, oxygen saturation.

**Table 2:** Hemodynamic parameters at beginning and at 30-minute of SBT.

**Hemodynamic Parameters**	**All patients (n=42)**	**Groups**		**P**-value
		**Successful Extubation (n=37)**	**Fail Extubation (n=5)**	
**At first minute of SBT,** **mean ± SD**				
Respiratory rate (breaths/min)	16.76±3.43	16.30±3.09	20.20±4.27	0.015^a^
Peripheral oxygen saturation (SaO_2_; %)	99.52±1.02	99.7±0.62	98.20±2.17	0.034^b^
MAP (mm Hg)	85.60±12.42	84.14±11.86	96.40±12.22	0.037^a^
Heart rate (beats/min)	88.20±16.40	86.86±16.57	98.40±11.84	0.142^a^
**At 30 minutes of SBT,** **mean ± SD**				
Respiratory rate (breaths/min)	18.48±3.70	17.97±3.35	22.20±4.71	0.015^a^
Peripheral oxygen saturation (SaO_2_; %)	99.55±1.04	99.76±0.49	97.80±2.34	0.034^b^
MAP (mm Hg)	88.29±11.90	86.86±11.39	98.80±12.13	0.037^a^
Heart rate (beats/min)	91.21±15.90	89.38±15.92	104.80±7.16	0.142^a^

**Note**: ^a^Independent T-test; ^b^Mann-Whitney-U test MAP, mean arterial pressure; SBT, spontaneous breathing trials.

**Table 3:** Blood gas analysis and ScvO_2_ level at beginning and at 30-minute of SBT.

**Blood Gas Analysis Parameters**	**All Patients (n=42)**	**Groups**		**P**-value
		**Successful Extubation (n=37)**	**Fail Extubation (n=5)**	
**At first minute of SBT, mean ± SD**				
pH	7.342±0.106	7.345±0.083	7.318±0.228	0.802^a^
pO_2_	41.07±7.61	40.27±5.91	47.00±15.13	0.379^a^
pCO_2_	43.84±6.81	44.62±6.61	38.08±5.88	0.042^a^
BE	-1.33±6.63	-1.03±5.32	-3.60±13.72	0.984^b^
HCO_3_	24.37±4.96	24.66±4.03	22.22±9.97	0.712^b^
ScvO_2_	70.74±8.22	70.00±7.76	76.20±10.40	0.167^b^
**At 30 minutes of SBT, mean ± SD**				
pH	7.344±0.082	7.345±0,072	7.335±0.152	0.811^a^
pO_2_	37.81±5.91	37.73±5.52	38.40±9.13	0.830^b^
pCO_2_	44.34±7.46	45.06±7.33	39.08±6.92	0.087^b^
BE	-1.19±5.88	-0.95±4.99	-3.00±11.20	0.470^a^
HCO_3_	24.34±4.89	24.64±4.28	22.12±8.57	0.037^a^
ScvO_2_	67.21±8.05	67.11±7.75	68.00±11.07	0.142^a^
**Change during SBT, mean difference ± SD**				
ΔpH	0.002±0.051	-0.0003 ±0.042	0.018±0.100	0.460^b^
ΔPO_2_	-3.26±3.96	-2.54±1.88	-8.6±9.42	0.016^a^
ΔPCO_2_	0.50±3.32	0.44±3.46	1.00±2.22	0.727^a^
ΔBE	0.14±2.01	0.08±1.86	0.60±3.13	1.000^b^
ΔHCO_3_	-0.03±1.73	-0.02±1.71	-0.10±2.06	0.928^a^
ΔScvO_2_	-3.52±2.67	-2.89±1.63	-8.20±4.27	0.049^a^

**Note**: ^a^Independent T-test; ^b^Mann-Whitney-U test BE, base excess; HCO_3_, bicarbonate; PCO_2_, partial pressure of carbon dioxide; PO_2_, partial pressure of oxygen; SD, standard deviation; ScvO_2_, central venous oxygen saturation.

**Table 4:** Cross-tabulation between ΔScvO_2_ cut-off and extubation outcome.

**ΔScvO_2_**	**Extubation Outcome**		**Total**
	**Successful Extubation**	**Fail Extubation**	
≤4.5%	30 (81,1%)	2 (40%)	32 (76,2%)
>4.5%	7 (18,9%)	3 (60%)	10 (23,8%)
Total	37 (59.1%)	18 (40.9%)	42 (100.0%)

**Note**: ΔScvO_2_, mean difference between first minute and after 30 minutes of SBT.

**Table 5:** Diagnostic analysis of ΔScvO_2_ cut-off.

**Variable**	**AUC**	**Sensitivity**	**Specificity**	**Cut-off Point**	**CI95%**
ΔScvO_2_	89.7%	81.1%	60%	-4.5	0.769 – 1.000

**Note**: AUC, area under the curve; CI95%, confidence interval 95%; ΔScvO_2_, mean difference between first minute and after 30 minutes of SBT.

## Data Availability

The data and supportive information are available within the article.

## LIST OF ABBREVIATIONS

PS: Pressure Support
COPD: Chronic Obstructive Pulmonary Disorder
ICU: Intensive Care Unit
RSBI: Rapid Shallow Breathing Index
SBT: Spontaneous Breathing Trial
